# Concurrent Metformin-Associated Lactic Acidosis and Euglycemic Diabetic Ketoacidosis Without Sodium-Glucose Cotransporter 2 Inhibitor Exposure: A Case Report and Literature Review

**DOI:** 10.7759/cureus.106919

**Published:** 2026-04-12

**Authors:** Motaz Almahmood, Leen Abuafifeh, Amin Saied Sanosi Saied, Abdalla Fadul, Ayah Al Qaryoute, Hatem Ahmed, Muhammad Ali Tariq

**Affiliations:** 1 Internal Medicine, Tower Health Medical Group, Phoenixville, USA; 2 Internal Medicine, Hamad Medical Corporation, Doha, QAT; 3 Internal Medicine, Hamad General Hospital, Doha, QAT; 4 Geriatrics &amp; Long-Term Care, Hamad General Hospital, Doha, QAT; 5 Physiology, Appalachian State University, Boone, USA

**Keywords:** diabetes mellitus type 2, euglycemia diabetic ketoacidosis, high anion gap metabolic acidosis, metformin, metformin-associated lactic acidosis, renal replacement therapy (rrt)

## Abstract

Metformin-associated lactic acidosis (MALA) and euglycemic diabetic ketoacidosis (euDKA) are distinct causes of high anion gap metabolic acidosis, but their simultaneous occurrence is rare and may be overlooked when severe hyperlactatemia dominates the presentation. We report the case of a 68-year-old man with type 2 diabetes mellitus treated with basal-bolus insulin, metformin, and linagliptin who presented with vomiting, confusion, fatigue, hypotension, and reduced level of consciousness. Initial evaluation revealed profound acidemia (arterial pH 6.75), severe high anion gap metabolic acidosis (anion gap 51 mEq/L, bicarbonate <2 mEq/L), marked hyperlactatemia (19 mmol/L), significant ketonemia (beta-hydroxybutyrate >9.60 mmol/L), near-normal glucose (9.1 mmol/L), hyperkalemia (6.5 mEq/L), and severe acute kidney injury (creatinine 516 µmol/L). Ongoing metformin exposure in the setting of acute kidney injury supported MALA, while marked ketonemia with near-normal glucose supported concurrent euDKA, likely precipitated by vomiting and reduced oral intake. The patient was treated with intravenous fluids, vasopressors, sodium bicarbonate, insulin with concurrent dextrose, and sustained low-efficiency dialysis, with subsequent hemodynamic, metabolic, and renal recovery. This case emphasizes that marked hyperlactatemia does not exclude a concurrent ketotic process and that early ketone measurement can materially change management in metformin-treated patients with persistent high anion gap acidosis.

## Introduction

Metformin-associated lactic acidosis (MALA) is an uncommon but potentially life-threatening complication that typically occurs in the setting of metformin accumulation, impaired renal clearance, tissue hypoperfusion, or a combination of these factors [1,2]. It is usually characterized by high anion gap metabolic acidosis with marked hyperlactatemia and may progress rapidly to hemodynamic instability, multiorgan dysfunction, and the need for renal replacement therapy [1,2]. In contrast, euglycemic diabetic ketoacidosis (euDKA) is defined by ketoacidosis with normal or only mildly elevated blood glucose levels, which can delay recognition because severe hyperglycemia is absent [3]. Although sodium-glucose cotransporter 2 inhibitor therapy is the most widely recognized precipitant, euDKA may also occur with prolonged fasting, reduced oral intake, vomiting, infection, pregnancy, or relative insulin deficiency [3,4].

The concurrent occurrence of MALA and euDKA is rare and poses a diagnostic challenge because severe hyperlactatemia may overshadow a second acid-generating process [2-4]. Failure to identify the ketotic component early may delay initiation of insulin and dextrose therapy, while delayed recognition of MALA may postpone renal replacement therapy in appropriate cases. We report a patient with type 2 diabetes mellitus who presented with profound high anion gap metabolic acidosis, acute kidney injury, marked hyperlactatemia, and significant ketonemia despite near-normal glucose levels, consistent with overlapping MALA and euDKA. This case highlights the importance of considering mixed metabolic emergencies in metformin-treated patients with persistent high anion gap acidosis and supports early ketone testing even when lactate is markedly elevated.

## Case presentation

A 68-year-old man with hypertension, coronary artery disease status post coronary artery bypass grafting more than 10 years earlier, Guillain-Barré syndrome with residual quadriparesis, and type 2 diabetes mellitus of more than 15 years’ duration presented with one day of confusion, vomiting, fatigue, and reduced level of consciousness. His outpatient diabetes regimen included basal-bolus insulin, metformin, and linagliptin. He was not taking a sodium-glucose cotransporter 2 inhibitor.

Before presentation, he had repeated episodes of vomiting and progressive drowsiness. There was no reported fever, cough, diarrhea, chest pain, or decreased urine output. His family noted hypotension and cold extremities at home. Emergency medical services administered 1 L of intravenous normal saline, and norepinephrine was started during transfer because of persistent hypotension.

On arrival to the emergency department, he remained tachycardic at 115 beats/min, hypotensive at 95/52 mmHg, tachypneic at 35 breaths/min, and afebrile. Additional intravenous crystalloid was administered, blood and urine cultures were obtained, empiric antibiotics were initiated while sepsis was being evaluated, and vasopressor support was escalated with the addition of vasopressin because of ongoing shock. Initial laboratory testing demonstrated severe acute kidney injury and profound high anion gap metabolic acidosis. Key admission values are shown in Table 1. Inflammatory markers were only modestly elevated, with a C-reactive protein level of 12.7 mg/L and a procalcitonin level of 1.09 ng/mL. Blood and urine cultures remained negative, antibiotics were subsequently discontinued, and chest radiography and abdominal ultrasonography were unrevealing.

**Table 1:** Admission laboratory values with reference ranges.

Parameter	Result	Units	Reference range
Creatinine	516	umol/L	62-106
Blood urea nitrogen	23.9	mmol/L	2.5-7.1
Potassium	6.5	mEq/L	3.5-5.0
Sodium	141	mEq/L	135-145
Chloride	88	mEq/L	96-106
Bicarbonate	<2	mEq/L	22-29
Anion gap	51	mEq/L	8-12
Arterial pH	6.750	--	7.35-7.45
Lactate	19	mmol/L	0.5-2.2
Beta-hydroxybutyrate	>9.60	mmol/L	<0.60
Glucose	9.1	mmol/L	3.9-7.8
Ethanol	5	mg/dL	0-50

The combination of ongoing metformin exposure, severe hyperlactatemia, profound acidemia, and acute kidney injury supported MALA. Marked beta-hydroxybutyrate elevation with near-normal glucose also supported concurrent euDKA. The patient was treated with intravenous sodium bicarbonate, aggressive supportive care, and intravenous insulin administered with concurrent dextrose to suppress ketogenesis while maintaining euglycemia. Because of severe acidemia, hyperkalemia, shock, and acute kidney injury, emergent sustained low-efficiency dialysis was initiated. A second dialysis session was required the following day because of persistent metabolic derangement. Metformin was discontinued on admission and was not restarted.

Vasopressor requirements gradually decreased as perfusion improved. The patient remained in the intensive care unit for five days, after which he stabilized without ongoing vasopressor support. By discharge, renal function and acid-base status had improved substantially, with a creatinine level of 102 µmol/L, blood urea nitrogen level of 6.5 mmol/L, potassium level of 3.6 mEq/L, anion gap of 10 mEq/L, lactate level of 1.2 mmol/L, beta-hydroxybutyrate level of 0.4 mmol/L, and arterial pH of 7.37. He was discharged on an insulin-based diabetes regimen and instructed to avoid future metformin use.

## Discussion

This case is clinically important because the very high lactate concentration could easily have been accepted as the sole explanation for the patient’s acidosis. However, the markedly elevated beta-hydroxybutyrate level, the absence of marked hyperglycemia, and the persistent high anion gap supported concurrent euDKA. The diagnostic challenge in such cases is that both MALA and euDKA can present with nausea, vomiting, tachypnea, shock, and high anion gap metabolic acidosis. Diagnostic anchoring on lactate alone may therefore delay insulin-based therapy, even when ketosis is contributing meaningfully to the acid burden [2-4].

The coexistence of both processes has important therapeutic implications. Severe MALA requires immediate discontinuation of metformin, aggressive supportive care, and consideration of extracorporeal treatment when there is profound acidemia, marked hyperlactatemia, shock, altered mental status, or significant kidney dysfunction [5]. The EXTRIP workgroup recommends extracorporeal therapy in severe metformin poisoning, and our patient met multiple high-risk features, including pH less than 7.0, shock, altered consciousness, hyperkalemia, and acute kidney injury [5]. More recent observational data also suggest that kidney replacement therapy, particularly hemodialysis-based modalities, may be associated with lower mortality in MALA with acute kidney injury, although these data remain nonrandomized [6,7]. For euDKA, the therapeutic cornerstone remains intravenous fluids, insulin infusion, electrolyte monitoring, and early dextrose administration to permit continued insulin delivery despite near-normal glucose [3,4]. In overlap syndromes, dialysis may improve lactate, potassium, and metformin clearance, but it does not replace the need for insulin-mediated suppression of ketogenesis.

The published literature on concurrent MALA and euDKA remains limited and consists mainly of one small case series and several individual case reports [8-14]. As summarized in Table 2, the reported cases share recurring features, including impaired metformin clearance due to acute kidney injury or advanced chronic kidney disease, reduced oral intake, vomiting, dehydration, and other physiologic stressors that promote ketosis. Across these reports, the diagnostic challenge is that severe hyperlactatemia may dominate the clinical picture and obscure concurrent ketoacidosis, particularly when blood glucose is normal or only mildly elevated.

**Table 2:** Previously reported adult cases of concurrent metformin-associated lactic acidosis and euglycemic diabetic ketoacidosis. MALA, metformin-associated lactic acidosis; euDKA, euglycemic diabetic ketoacidosis; ICU, intensive care unit.

Report	Clinical context	Precipitating factors/exposure	Diagnostic clues	Management and outcome
Schwetz et al., 2017 [8]	Three ICU patients receiving metformin; renal failure in all three; two had no sodium-glucose cotransporter 2 inhibitor exposure	Renal failure with reduced metformin clearance; fasting-related ketosis proposed	Glucose 63-150 mg/dL with concurrent severe lactatemia and ketoacidosis	Glucose infusion plus renal replacement therapy; all improved
Hasnie et al., 2021 [9]	Metformin-treated adult presenting with neurologic symptoms and persistent high anion gap acidosis	Metformin exposure; overlap recognized after lactate began to improve	Ketonuria and persistent anion gap despite improvement in lactate	Sodium bicarbonate followed by insulin with dextrose; anion gap resolved
Kuno et al., 2023 [10]	Adult with metformin/vildagliptin overdose, acute kidney injury, vomiting, and stress physiology	Intentional overdose with impaired renal clearance	Lactate decreased after hemodialysis, but persistent anion gap and ketonemia revealed euDKA	Single hemodialysis session; recovery
Nzomessi et al., 2023 [11]	Woman with severe heart failure treated with metformin and empagliflozin	Fasting, acute kidney injury, and combined metformin plus sodium-glucose cotransporter 2 inhibitor exposure	Progressive euDKA complicated by MALA and acute renal failure	Intermittent hemodialysis; recovery
Shiplett and Mathias, 2024 [12]	68-year-old woman with end-stage renal disease on metformin who had missed dialysis; no sodium-glucose cotransporter 2 inhibitor	Missed dialysis, nausea, vomiting, and markedly impaired metformin clearance	Lactate >20 mmol/L, beta-hydroxybutyrate >8 mmol/L, glucose 121 mg/dL	Bicarbonate, insulin, and continuous renal replacement therapy; discharged on basal-bolus insulin
Tomita et al., 2025 [13]	Woman in her 50s with chronic heart failure receiving metformin and a sodium-glucose cotransporter 2 inhibitor	Heat-related dehydration and fluid restriction	Hemodialysis lowered lactate, but persistent anion gap and elevated beta-hydroxybutyrate confirmed euDKA	Hemodialysis followed by insulin infusion; recovery
Hussein et al., 2025 [14]	63-year-old woman on metformin and insulin with gastrointestinal illness, shock, and acute kidney injury	Gastroenteritis, dehydration, and multiorgan dysfunction	Profound acidemia with euDKA, severe lactatemia, and cardiac arrest	Continuous venovenous hemodiafiltration, vasopressors, and ventilation; full recovery

Taken together, the published cases reinforce the same diagnostic lesson: severe hyperlactatemia should not be assumed to fully explain high anion gap metabolic acidosis in a metformin-treated patient. Persistent ketonemia, ketonuria, or an anion gap that remains wider than expected after partial lactate improvement should prompt active evaluation and treatment for concurrent euDKA [8-14]. Although not a metformin-related overlap syndrome, other mixed metabolic emergencies have also been reported. Almahmood et al. described olanzapine-induced diabetic ketoacidosis occurring concurrently with neuroleptic malignant syndrome, underscoring the risk of diagnostic anchoring when one dramatic presentation obscures a second life-threatening process [15].

A practical limitation in diagnosing MALA is that serum metformin concentrations are rarely available in real time and are not required for bedside treatment decisions [16]. In our patient, the combination of ongoing metformin exposure, severe acute kidney injury, profound acidemia, marked hyperlactatemia, and subsequent improvement after dialysis made the diagnosis clinically persuasive.

In this case, recurrent vomiting and reduced oral intake were the most plausible precipitants of ketosis, whereas the absence of sodium-glucose cotransporter 2 inhibitor exposure made medication-related euDKA less likely. Our patient also had severe acute kidney injury, ongoing metformin exposure, profound acidemia, and marked beta-hydroxybutyrate elevation, all of which supported the coexistence of both processes rather than isolated lactic acidosis alone.

The main limitation of this report is that the diagnosis of overlapping MALA and euDKA was made on clinical grounds rather than by a rapidly available serum metformin concentration. Nevertheless, the convergence of ongoing metformin use, acute kidney injury, severe hyperlactatemia, marked ketonemia, mild hyperglycemia, and resolution after dialysis plus insulin-based therapy makes the combined diagnosis persuasive.

## Conclusions

Concurrent MALA and euDKA should be considered in metformin-treated patients who present with severe high anion gap metabolic acidosis, particularly when acute kidney injury, vomiting, dehydration, or reduced oral intake is present. Marked hyperlactatemia does not exclude a second acid-generating process. Early ketone measurement, prompt insulin with dextrose, and timely extracorporeal therapy when indicated may improve recognition and management of this uncommon but potentially life-threatening overlap syndrome.
